# Parametrically Optimized Carbon Nanotube-Coated Cold Cathode Spindt Arrays

**DOI:** 10.3390/nano7010013

**Published:** 2017-01-12

**Authors:** Xuesong Yuan, Matthew T. Cole, Yu Zhang, Jianqiang Wu, William I. Milne, Yang Yan

**Affiliations:** 1 School of Physical Electronics, University of Electronic Science and Technology of China, Chengdu 610054, China; jqwu@uestc.edu.cn (J.W.); yanyang@uestc.edu.cn (Y.Y.); 2 Electrical Engineering Division, Department of Engineering, University of Cambridge, Cambridge CB3 0FA, UK; mtc35@cam.ac.uk (M.T.C.); wim@eng.cam.ac.uk (W.I.M.); 3 State Key Laboratory Optoelectronic Materials and Technologies, Sun Yat-sen University, Guangzhou 510275, China; stszhyu@mail.sysu.edu.cn

**Keywords:** carbon nanotube, cold cathode, field emission, electron gun

## Abstract

Here, we investigate, through parametrically optimized macroscale simulations, the field electron emission from arrays of carbon nanotube (CNT)-coated Spindts towards the development of an emerging class of novel vacuum electron devices. The present study builds on empirical data gleaned from our recent experimental findings on the room temperature electron emission from large area CNT electron sources. We determine the field emission current of the present microstructures directly using particle in cell (PIC) software and present a new CNT cold cathode array variant which has been geometrically optimized to provide maximal emission current density, with current densities of up to 11.5 A/cm^2^ at low operational electric fields of 5.0 V/μm.

## 1. Introduction

In the drive towards realizing the next-generation of high performance electron emission systems for use in vacuum devices, carbon nanotubes are coming to the fore as a noteworthy material. They readily overcome many of the intrinsic limitations associated with incumbent and pervasive thermionic devices, including functional issues associated with their high temperature operation and slow temporal response [1,2,3,4,5]. By manufacturing via advanced chemical vapor deposition (CVD) processes [6,7], inexpensive nanostructured cold cathode electron sources can be obtained and are capable of improved operation relative to traditional Spindt-type arrays. Nevertheless, there remains a significant challenge to simultaneously producing high currents and high current densities across large areas from such nanostructured devices. 

Simple consideration of the field enhancement factor alone has repeatedly proven insufficient in optimizing the emission architecture [8,9]. Further detailed consideration of the interplay in the emission geometry and the emission performance will likely manifest in functional enhancements and improved manufacturability. In designing carbon nanotube (CNT) vacuum electronic devices, it is important that such design tools take into consideration the real emission characteristics of typical CNT cold cathodes. A truly nanometrically resolved, macroscale model would certainly be computationally infeasible and overly time consuming in its processing. Nevertheless, there remains merit in producing computational tools capable of approximating the emission from such multi-scalar systems. 

An equivalent field emission model of CNT-coated Spindts is proposed here, which we hope will prove to be the basis of the development of an emerging class of CNT-based vacuum electronic devices. We present a method to build a CNT-based cold cathode field emission model using experimental findings. Field emission equivalent parameters are calculated by numerical fitting. For various experimental CNT structures, we build a corresponding field emission simulator whose equivalent parameters in a particle in cell (PIC) simulation are subsequently parametrically optimized. Optimal architectures are therein investigated based on the suggested geometries with the present study offering a concise means of modeling the emission characteristics from CNT-based electron gun systems for future vacuum device development.

## 2. CNT Cold Cathode Simulator

During field electron emission, many thousands of tips concurrently emit with individual tips themselves notably having multiple emission sites [10]. In the case of the former, the unavoidable variation in CNT geometries within a particular sample induces variation within the spatial and temporal emission characteristics. It is highly improbable that all the nanometer tips emit to the same extent simultaneously. Thus, it is evidently critical in building a model that accurately conveys this distinct lack of unavoidable sub-micronscale homogeneity. Most simulation packages include integrated field emission models based on electron rich metallic and quasi-metallic materials. Such emission is readily described by the simplified Fowler–Nordheim (FN) equation: *J* = *A* × *E* × *exp*(−*B*/*E*), where *A* and *B* are the simplified Fowler–Nordheim constants. If *A* and *B* are expressly obtained for an equivalent, metallic, or quasi-metallic CNT field emission system through experimentation, we can use this to model, to modest accuracy, a variety of CNT-based emission architectures based on intrinsically integrated empirical findings. Across a variety of measured samples, non-linear least-squares fitting was adopted to determine *A* and *B*. In order to do this, we firstly selected appropriate experimental models which satisfy two central conditions. Firstly, edge effects must be reduced to an absolute minimum to best capture the emission properties of an otherwise infinite CNT array, since edge effects dramatically adjust the emission characteristics. Secondly, our fitted constants were extracted from experimental results from a large number of emitters in order to give an accurate equivalent model that represents an average emission performance of a large CNT population. 

**Figure 1:**
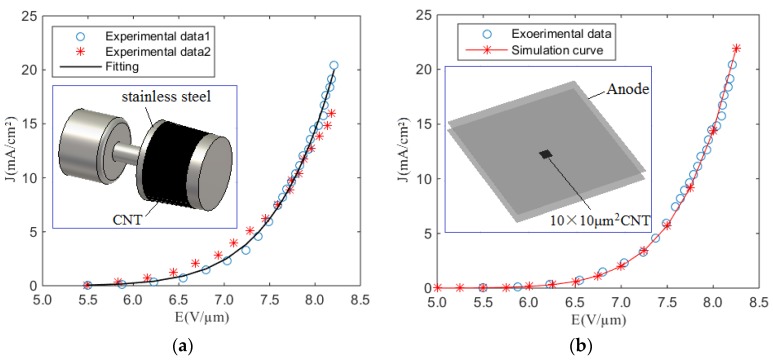
Fitting, simulation, and experimental results. (**a**) Fitting and experimental current density as a function of global electric field. Inset: Scheme depicting the truncated-Spindt carbon nanotube cold cathode electron source; (**b**) Simulation and experimental for a generalized square emitter confirming the commutability of the Fowler–Nordheim (FN) coefficient extraction processed outlined. Inset: simulation geometry.

Based on the extracted coefficients, a truncated-cone carbon nanotube cold cathode was developed as a model system, as reported in detail elsewhere [11]. Here, CNTs were grown directly on the sidewalls of a stainless steel truncated-cone by thermal chemical vapor deposition, as illustrated in the inset of Figure 1a. This CNT-coated cone was subsequently sandwiched between two additional hollow stainless steel, nested, truncated-cones to form the emission cavity. The inner wall of the anode lies parallel to the outer wall of the cathode in a coaxial structure. The cathode height was 9.8 mm. The top and bottom radii were 8.0 mm and 8.5 mm, respectively. The total cathode area was 5.1 cm^2^. Typical emission spectra of two truncated-cone carbon nanotube cathodes, operated in diode mode, are shown in Figure 1a [12], with all experiments suggesting a maximum emission current of 103 mA. Applying our fitting procedure we find *A* = 3.41 × 10^−7^ A/V^2^ and *B* = 9.55 × 10^7^ V/m. Figure 1a shows typical experimental data and the resulting fitted emission characteristics. A good fit is noted (*R*^2^ = 0.994). Using the extracted *A* and *B* in our PIC simulations, our models suggest an equivalent electric field dependent emission with less than a 1% deviation between the model and the experimental data, for all electric fields considered in this study. To confirm the validity of the approach, we applied the extracted coefficients to a further arbitrary geometry and compared the simulation to the latterly produced empirical findings. Figure 1b shows typical simulated and experimental emission current density profiles as a function of anode voltage. Here, we use a 10 × 10 μm^2^ square emission area consisting of nominally equivalent CNTs throughout. The planar inter-electrode gap was set to 10 µm. We find good agreement between our simulation and experimental data with a general invariance with the size of the inter-electrode gap in the far field. We stress that the extracted *A* and *B* coefficients are not general parameters but rather material specific values that are characteristic of the broader CNT family. They are specific to the CNTs under study and are intimately related to the CVD method of manufacture and the growth environment therein. Based on this method, different electron guns using such CNTs can be developed with reduced concerns on the detailed growth characteristics in an attempt to ultimately decouple the growth and vacuum device development activities [13,14].

## 3. Cathode Optimization

A key function of the present simulator is to optimize the emitter geometries. To improve the emission current, we must take into account both the field enhancement factor and the emitter’s propensity towards emission per unit area. In general, the field enhancement factor of a typical Spindt-type cathode or vertical grown individual CNT is very large whilst the size of the emitting tips is very small [15,16]. Using such geometries, we can dramatically reduce the operational electric field. However, given the low emitted current per tip, in order to obtain the high emission currents required by a variety of vacuum devices, it is necessary to manufacture thousands of emitter tips over comparatively large areas. Ensuring growth uniformity across the entire emitter population remains challenging, with any variations in emitter height being a largely random occurrence that cannot be modeled with any fidelity. The use of empirically defined constants in the present study makes it possible to obviate such issues in so far as these constants intrinsically integrate such morphological, and hence emission, irregularities. Growth disorder, which manifest repeatedly at the macroscale, becomes implicitly integrated into our modeling efforts. This simplifies the otherwise demanding computational complexity dramatically. As a result, such modeling tools will allow for the widespread use of thermal and plasma enhanced chemical vapor deposition as a means of nano-fabrication with reduced concerns over microscale and sub-microscale growth uniformity. Nevertheless, the field enhancement factors associated with such CNT forests is limited, and low, since the spacing between adjacent CNTs is small. The CNTs experience severe nearest neighbor electrostatic shielding. An optimized emission geometry would combine the relative merits of Spindt-type sources with those of CNT thin films to produce a concurrently high current and current density. Making use of Spindts increases manufacturability, whilst the CNTs ensure a high emission current per unit area. Such CNT-coated Spindt-type emitters have been realized elsewhere [17,18], with the system benefitting from the low voltage field emission of the CNTs coupled to the large area, highly reproducible fabrication of the micronscale Spindts. Nevertheless, the geometry of such CNT-coated Spindt arrays must be optimized in order to satisfy various system requirements.

### 3.1. The Single Spindt Model

**Figure 2:**
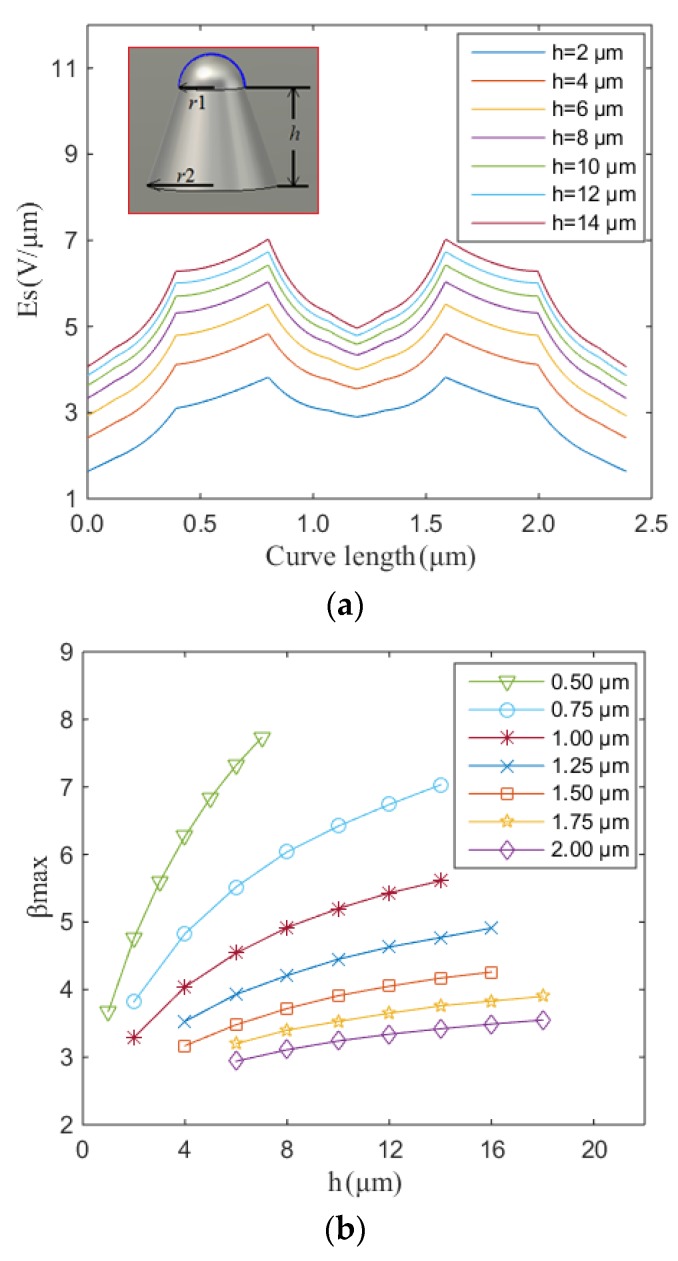

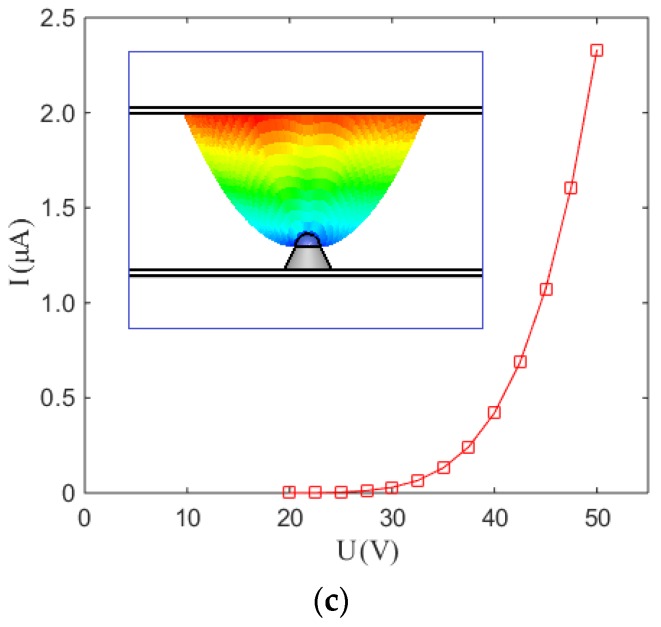
Single carbon nanotube (CNT)-coated Spindt field emission. (**a**) Surface electric field as a function of Spindt height. Inset: single Spindt geometry. (**b**) The maximum field enhancement factor β_max_, extracted from the geometry terms, as a function of *h* and *r*_1_. (**c**) Simulated emission from a single Spindt CNT cold cathode. Inset: beam trajectories.

In order to abstract this otherwise highly parallelized emission system, we first broach the field enhancement factor of a single Spindt-like CNT-coated cold cathode. The inset of Figure 2a depicts the equivalent single Spindt geometry. Here, there is a single Spindt on an otherwise large (near infinite) plate (cathode) lying parallel to a planar anode. The Spindt tip is assumed to be, to the first order, hemispherical. The bottom radius *r*_2_ is set to twice the tip radius *r*_1_. The height of the Spindt is *h*. The distance *d* between the anode and the top of the Spindt is 10 μm. The blue line on the surface of the hemisphere, in the inset of Figure 2a, denotes the probed surface under study. Figure 2a shows the electric field distribution along the blue line as a function of height of the Spindt. Here, *r*_1_ is 0.75 μm. The cathode and anode bias are 0 V and 10 V, respectively. In the case of an infinite parallel plate of equivalent potential difference, the electric field *E_p_* is 1 V/μm. However, the maximum surface electric field on the Spindt, as a function of height, is seen to increase from 3.8 V/μm to 6.4 V/μm given the notable geometry mediated field enhancement. The average geometry-based enhancement factor (<β> = *E_s_/E_p_*) is >5 when *h* = 10 μm. Figure 2b shows the variation in the maximum field enhancement factor β_max_ with *h* and *r*_1_. When the height increases, the maximum field enhancement factor increases. When the radius increases, the maximum field enhancement factor decreases. When *r*_1_ is >1.50 μm, the variation in the maximum field enhancement factor with height changes only marginally. After obtaining the field enhancement factor, the field emission current can then be calculated using PIC software (CST particle studio, Darmstadt, Germany). Compared with the electric field denoted on the cross-sectional analysis in Figure 2a, the electric field on the side wall of the Spindt is very low. The emission was dominated, as expected, by the top hemisphere of the Spindt. Here, *r*_1_ = 1.00 μm and *h* = 2.00 μm, giving a β_max_ = 3.3, as shown in Figure 2c.

### 3.2. Array Optimization

**Figure 3:**
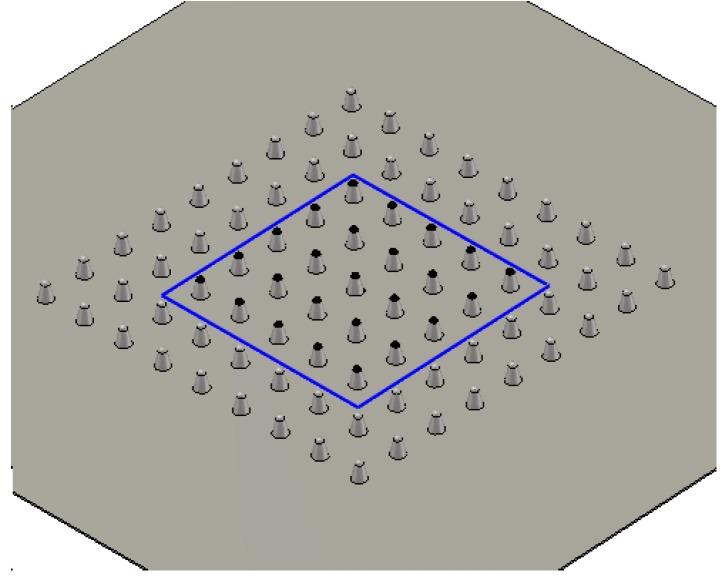
Simulated CNT-coated Spindt cold cathode array.

Based on the above single Spindt simulation, a CNT cold cathode array has been investigated herein. Figure 3 shows one possible model of a CNT-coated Spindt array. A square 9 × 9 array is set on the lower plate (cathode). To obviate edge effects, only the central 5 × 5 square array considered. CNTs are conformably grown exclusively on the upper most surfaces of the Spindts. The distance between two individual Spindts is *D*. All other parameters remain the same as per the earlier single Spindt model. The emission current was first obtained through PIC simulation. The emission current density was estimated using *J = I/S = I/*(5*D* × 5*D*), where *S* denotes the present square array. 

There are four dominant factors influencing the level of field emission in the optimization; the emitter tip radius *r*, emitter height *h*, inter-emitter pitch *D*, and the global electric field *E*. Here, *r*, *h*, and *D* are central in influencing the field enhancement factor β. *E* and β determine the surface electric field *E_s_*, which dictates the emission current density *J*. Given experimental limitations; we set a maximum global electric field (5 V/μm) and a surface electric field (20 V/μm) in the optimization. Taking a gridded electron gun as an example, the typical height of emitter is of the order of 5–10 μm. The distance between the anode (grid) and cathode is usually larger than 0.2 mm, which is >> *h* in order to reduce arcing affects associated with such height differences. Supposing that the drop in electric field beneath the center of grid is 50%, as evidenced elsewhere [14], we must then set the grid electrode to 2 kV to obtain a global electric field of 5 V/μm. Upon transmission through the grid, electron energies are maximally 2 keV, with an increasing velocity dispersion. Focusing of the electron beam becomes challenging. The maximum surface electric field was determined through vacuum breakdown experiments of individual CNTs [19]. When the electric field on surface of CNT is larger than 20 V/μm, significant vacuum breakdown occurs. 

**Figure 4:**
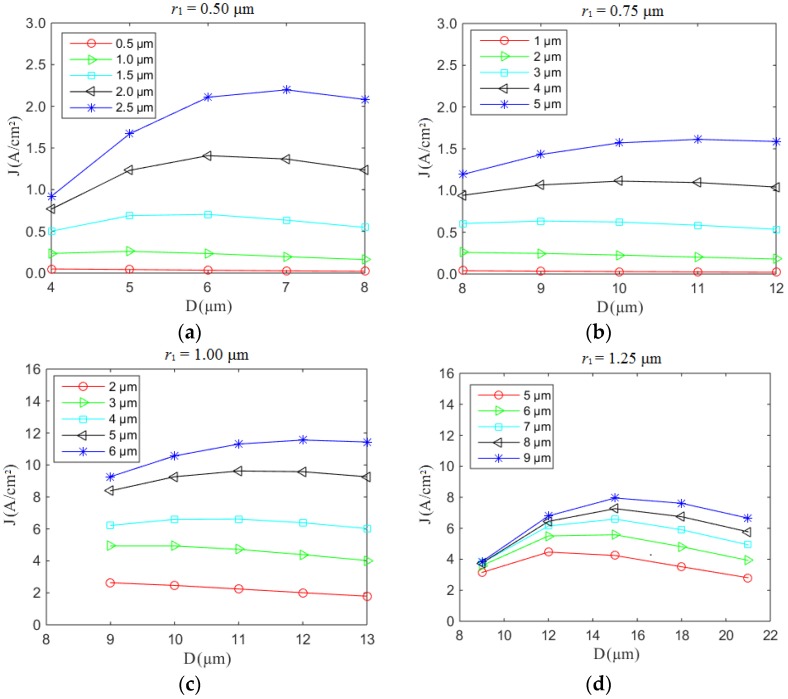
Field emission current density as a function of *h* and *D*. Under low-bias (35 V), (**a**) *r*_1_ = 0.50 μm and (**b**) *r*_1_ = 0.75 μm; under high-bias (50 V), (**c**) *r*_1_ = 1.00 μm and (**d**) *r*_1_ = 1.25 μm.

As noted above, as *r*_1_ increases the maximum field enhancement factor decreases dramatically. For brevity’s sake, we consider four practically viable *r*_1_ values (0.50, 0.75, 1.00, and 1.25 μm) in the present geometry. An equivalent global electric field of 3.5 V/μm is used for *r*_1_ = 0.50 and 0.75 μm. The maximum electric field on the surface set to <20 V/μm. A maximum field enhancement factor of ~5.7 is obtained. These parameters were selected to ensure that the height-to-radius ratio (η = *h*/(2 × *r*_2_)) is commensurate with our empirical findings (Figure 2b). Though the larger η is the better, it becomes increasingly difficulty to experimentally produce such high aspect ratios of ever-narrower diameters at maintained or increased heights. Figure 4a shows the field emission current density as a function of *h* and *D* for a radius of 0.50 μm. The resulting maximum current density of 2.2 A/cm^2^ compares favorably with the state of the art in field electron emission sources [20]. Upon geometrical optimization, the current density is seen to increase by two orders of magnitude compared with our experimental results. The global electric field decreases from 8.3 V/μm to 3.5 V/μm. As has been highlighted elsewhere [21], as *h* increases, the emission current density increases. Figure 4b shows the emission current density with a radius of 0.75 μm. Compared with Figure 4a,b, a larger emission current density is readily obtained under the same electric field when the top radius of the Spindt decreases. The emission current density reaches a maximum when the distance *D* is approximately three times the height *h*. When the radius *r*_1_ increases to >1.00 μm, the maximum field enhancement factor decreases obviously, as shown in Figure 2b. As a result, an equivalent global electric field of 5 V/μm is used to achieve a surface electric field of 20 V/μm. Figure 4c shows the field emission current density as a function of *h* and *D* for a radius of 1.00 μm. The maximum emission current density is 11.5 A/cm^2^, which is larger than that for an electric field of 3.5 V/μm. In Figure 4d, the maximum emission current density is 8 A/cm^2^, and the field enhancement factor is 4.3.

## 4. Discussion

In order to improve the field emission current and current density simultaneously, a CNT-coated Spindt cold cathode has been investigated here via parametric simulation based on optimized geometries. Although the emission is dominated by the tip of the CNT-coated Spindts in our model, in the present study, the CNTs are experimentally realized on all Spindt surfaces given the conformity of the catalyst physical vapor deposition and the CNT chemical vapor deposition. Our studies suggest that the inclusion of such nano ad-layers have almost no negative effects on the electron emission performance. Conservative by design, the present simulations produce a lower bound to the actual emission performance of the studied multi-component, multi-scalar devices. Our simulations also suggest that such systems give rise to increasingly diffuse beam trajectories that would likely induce significant grid interception losses that must be taken into consideration upon system design. One possible solution here would be to adopt a dual-array approach coupled to magnetic field steering, though this falls outside the scope of the present study. We have successfully fabricated a dual-array at both the macro- and micro-scales and are continuing to pursue the latter—We will report those findings elsewhere.

## 5. Conclusions

Carbon nanotubes are one of the leading candidate materials for the next generation of vacuum electronic devices. Their unique structure, growth, and electronic properties all near-ideally lend themselves to the fabrication of otherwise novel electron gun technologies. Therein, the development of a concise means of modeling such CNT cold cathode devices is of central importance for the optimized design of novel electron optical systems and advanced high-energy light sources. However, the changeable and often poorly described geometry of CNTs and ensembles based thereon make it difficult to accurately establish suitable emission geometries upon which to base simulations. Based on experimental data, we report here on an effective method to establish an equivalent model. Through a fitting of experimental data, an equivalent CNT field emission model is obtained, which we find accurately captures the emission characteristics under a variety of arbitrary cathode geometries. As the field emission current can be gleaned directly through such studies, this method has proven accurate compared to other more conventional field analysis methods. Based on this, a CNT-coated Spindt-like cathode has been optimized. Emission current densities of up to 2.2 A/cm^2^ have been attained at global electric fields as low as 3.5 V/μm. For global electric fields of 5 V/μm, the maximum current density reached 11.2 A/cm^2^. Based on this method, a new equivalent field emission model based on parametrically optimized simulation results has been obtained, aiding in the design of CNT cold cathode electron optical systems for advanced electron beam applications. 

## References

[1] Collins C.M., Parmee R.J., Milne W.I., Cole M.T. (2016). High performance field emitters. Adv. Sci..

[2] Milne W.I., Teo K.B.K., Minoux E., Groening O., Gangloff L., Hudanski L., Schnell J.-P., Dieumegard D., Peauger F., Bu I.Y.Y. (2006). Aligned carbon nanotubes/fibers for applications in vacuum microwave amplifiers. J. Vac. Sci. Technol. B.

[3] Kim H.J., Choi J.J., Han J.-H., Park J.H., Yoo J.-B. (2006). Design and field emission test of carbon nanotube pasted cathodes for traveling wave tube applications. IEEE Trans. Electron Devices.

[4] Ulisse G., Brunetti F., Tamburri E., Orlanducci S., Cirillo M., Terranova M.L., di Carlo A. (2013). Carbon Nanotube Cathodes for Electron Gun. IEEE Electron Device Lett..

[5] Manohara H.M., Toda R., Lin H.R., Liao A., Bronikowski M.J., Siegel P.H. (2009). Carbon nanotube bundle array cold cathodes for THz vacuum tube sources. J. Infrared Millim. Terahertz Waves.

[6] Cole M.T., Milne W.I. (2013). Plasma Enhanced chemical vapour deposition of horizontally aligned carbon nanotubes. Materials.

[7] Xu N.S., Chen J., Deng S.Z., She J.C. (2004). The Preparation Method of Carbon Nanotubes Film on Stainless Steel Substrate. China Patent.

[8] Bocharov G.S., Eletskii A.V. (2013). Theory of Carbon nanotube (CNT)-based electron field emitters. Nanomaterials.

[9] Berdinsky A.S., Shaporin A.V., Yoo J.-B., Park J.-H., Alegaonkar P.S., Han J.H., Son G.H. (2006). Field enhancement factor for an array of MWNTs in CNT paste. Appl. Phys. A.

[10] Saitoa Y., Uemurab S. (2000). Field emission from carbon nanotubes and its application to electron sources. Carbon.

[11] Yuan X., Zhu W., Zhang Y., Xu N., Yan Y., Wu J., Shen Y., Chen J., She J., Deng S. (2016). A Fully-sealed carbon-nanotube cold-cathode terahertz gyrotron. Sci. Rep..

[12] Yuan X., Zhang Y., Yan Y., Li X., Wu J., Deng S. Carbon nanotube magnetron injection electron gun for a 0.22THz gyrotron. Proceedings of the IEEE International Vacuum Electronics Conference (IVEC).

[13] Yuan X., Zhang Y., Yang H., Li X., Xu N., Deng S., Yan Y. (2015). A Gridded high-compression-ratio carbonnanotube cold cathode electron gun. IEEE Electron Device Lett..

[14] Yuan X., Wang B., Cole M.T., Zhang Y., Deng S., Milne W.I., Yan Y. (2016). Theoretical Research on a multibeam-modulated electron gun based on carbon nanotube cold cathodes. IEEE Trans. Electron Devices.

[15] Whaley D.R., Duggal R., Armstrong C.M., Bellew C.L., Holland C.E., Spindt C.A. (2009). 100 W Operation of a cold cathode TWT. IEEE Trans. Electron Devices.

[16] Cole M.T., Teo K.B.K., Groening O., Gangloff L., Legagneux P., Milne W.I. (2014). Deterministic cold cathode electron emission from carbon nanofibre arrays. Sci. Rep..

[17] Shahi M., Gautam S., Shah P.V., Rawat J.S., Chaudhury P.K., Harsh, Tandon R.P. (2013). Decoration of cesium iodide nano particles on patterned carbon nanotube emitter arrays to improve their field emission. J. Nanopart. Res..

[18] Gautier L., Le Borgne V., Al Moussalami S., El Khakani M.A. (2014). Enhanced field electron emission properties of hierarchically structured MWCNT-based cold cathodes. Nanoscale Res. Lett..

[19] She J.C., Xu N.S., Deng S.Z., Chen J., Bishop H., Huq S.E., Wang L., Zhong D.Y., Wang E.G. (2003). Vacuum breakdown of carbon-nanotube field emitters on a silicon tip. Appl. Phys. Lett..

[20] Li C., Zhang Y., Mann M., Hasko D., Lei W., Wang B., Chu D., Pribat D., Amaratunga G.A., Milne W.I. (2010). High emission current density, vertically aligned carbon nanotube mesh, field emitter array. Appl. Phys. Lett..

[21] Li Z., Yang X., He F., Bai B., Zhou H., Li C., Dai Q. (2015). High current field emission from individual nonlinear resistor ballasted carbon nanotube cluster array. Carbon.

